# Dose-response relationship in cisplatin-treated breast cancer xenografts monitored with dynamic contrast-enhanced ultrasound

**DOI:** 10.1186/s12885-015-1170-8

**Published:** 2015-03-17

**Authors:** Yao Chen, Feng Han, Long-hui Cao, Cheng Li, Jian-wei Wang, Qing Li, Wei Zheng, Zhi-xing Guo, An-hua Li, Jian-hua Zhou

**Affiliations:** 1 Department of Ultrasound, Sun Yat-Sen University Cancer Center, State Key Laboratory of Oncology in South China, Collaborative Innovation Center for Cancer Medicine, Guangzhou, 510060 People’s Republic of China; 2 Department of Anesthesiology, Sun Yat-Sen University Cancer Center, State Key Laboratory of Oncology in South China, Collaborative Innovation Center for Cancer Medicine, Guangzhou, 510060 People’s Republic of China

**Keywords:** Contrast-enhanced ultrasound, Perfusion, Cisplatin, Chemotherapy, Cancer

## Abstract

**Background:**

Exactly assessing tumor response to different dose of chemotherapy would help to tailor therapy for individual patients. This study was to determine the feasibility of dynamic contrast-enhanced ultrasound (CEUS) in the evaluation of tumor vascular response to different dose cisplatin.

**Methods:**

MCF-7 breast cancer bearing mice were treated with different dose of cisplatin in group B (1 mg/kg) and group C (3 mg/kg). A control group A was given with saline. Sequential CEUS was performed on days 0, 3 and 7 of the treatment, in which time-signal intensity curves were obtained from the intratumoral and depth-matched liver parenchyma. Peak enhancement (PE), area under the curve of wash-in (WiAUC), wash-in rate (WiR) and wash-in perfusion index (WiPI) were calculated from perfusion time-intensity curves and normalized with respect to the adjacent liver parenchyma. Histopathological analysis was conducted to evaluate tumor cell density and microvascular density (MVD).

**Results:**

Significant decreases in tumor normalized perfusion parameters were observed on day 3 in the high dose group and on day 7 in the low dose group. On day 7, nPE, nWiAUC, and nWiPI significantly decreased in group C and group B as compared with group A (*P <* 0.05), and further decreased in group C as compared with group B (*P* < 0.05). Significant decreases of tumor cell density and MVD were seen in treated group (group B and C) compared to control group (*P* < 0.05) and further decrease in group C compared to group B (*P* < 0.05).

**Conclusions:**

Dynamic CEUS for quantification of tumor perfusion could be used to evaluate tumor vascular response to different dose of chemotherapy.

## Background

The cytotoxic chemotherapeutics have been used for systemic cancer therapy for half a century. On the basis of the concept that a given dose of an antineoplastic compound would destroy a certain fraction of tumor cells, the efficiency of tumor chemotherapy, to a great extent, is dependent on the dosage of drugs and higher doses are supposed to be more effective. Therefore, high-dose chemotherapy with autologous bone-marrow transplants has been widely used in the treatment of rare types of cancer, unresponsive cancer and cancer relapse [1,2]. However, high-dose chemotherapy in solid tumor in order to boost the effect of anti-cancer therapy is always under controversy. The studies of Leonard RC and Roche H et al. showed that high-dose chemotherapy with autologous bone-marrow stem-cell transplants had not improved the overall survival and disease free survival as expected, when used for patients with metastatic breast cancer [3,4]. Moreover, this approach is also very expensive and highly toxic. Thus, exactly assessing tumor response to different dose of chemotherapy is urgently needed, which could not only be helpful to tailor therapy for individual patients, but also avoid wasting of medical resource.

Ultrasound is an attractive imaging technique for assessment tumor response to treatment because it can be repeated without exposing the patient or animal to any risk of radiation. Ultrasound imaging systems are also relatively inexpensive and mobile when compared other imaging modalities, a particular benefit for animal studies. The use of microbubble-based contrast agents combined with contrast specific imaging modalities has considerably improved the visualization of microvascular perfusion that is undetectable with traditional Doppler techniques. Noninvasive contrast-enhanced ultrasound imaging offers the major advantages of evaluating tumor perfusion in real time and without any risk of ionizing radiation when compared to other methods used to assess tumor perfusion [5]. Numerous studies have used dynamic contrast-enhanced ultrasound to assess vascular changes associated with response to antiangiogenic therapy [6-9]. Recently, the treatment effect of cytotoxic chemotherapy was evaluated with the use of dynamic contrast-enhanced ultrasound in both animal [10,11] and clinical studies [12,13] and preliminary results were promising.

The purpose of our study was to measure the dose–response relationship in cisplatin-treated breast tumor xenografts by using dynamic contrast-enhanced ultrasound.

## Methods

### Animal preparation

This study was approved by the Committee on the Ethics of Animal Experiments of the Sun Yat-Sen University under the guidelines of the National Institutes of Health for the care of laboratory animals. Human breast cancer cell line MCF-7 was obtained from State Key Laboratory of Oncology in Southern China. MCF-7 cells were grown in DMEM culture medium (Hyclone Co., UT, USA) supplemented with 10% fetal bovine serum (Gibco, Grand Island, NY, USA), penicillin (50U/ml), and streptomycin (50 μg/ml) at 37°C in a humidified 5% CO_2_ atmosphere. For inoculation, approximately 5 × 10^7^ MCF-7 cells suspended in phosphate-buffered saline were injected subcutaneously into the right flanks of 8-week old BALB/c female nude mice.

### Experimental design

A total of 70 mice were used for the experiment and the mice were randomly divided into 3 groups with group A (n = 26) as control subjects and groups B and C (n = 22 on each) as treatment subjects. Cytotoxic chemotherapy agent, cisplatin (Mayne Pharma Pty Ltd, Salisbury, Australia) diluted in sterile saline was administered by intraperitoneal injection once daily at the dose of 1 mg/kg for group B and 3 mg/kg for group C 12 days post tumor cell implantation. The mice in control group (group A) received vehicle control medium (sterile saline) with same timing and dosing schedule used for treatment group. The fluid volume of intraperitoneal injection given for the treatment and control tumors was 10 μl per gram of body weight. The time point for the first dose given was referred to as day 0. Ultrasound imaging was performed at days 0, 3 and 7 before each dosing. On day 0, 5 mice randomly chosen from the control group were sacrificed and tumors were excised for histopathologic analysis. On day 3, histopathologic analysis were performed in 9 mice of group A, 8 mice of group B and 8 mice of group C, respectively. On day 7, the remaining mice (11 mice in group A, 13 mice in group B and 14 mice in group C) were sacrificed and tumors were excised for histopathologic analysis after ultrasound imaging.

### US imaging protocol

One radiologist (with 8 years experience) who was blinded to treatment groups performed contrast-enhanced US for all groups on days 0, 3 and 7 before dosing of cisplatin. Ultrasound imaging with contrast pulse sequence (CPS) technique [14,15] were obtained using an Acuson Sequoia 512 (Siemens, Mountain View, CA) ultrasound unit with a linear array transducer (7.0 ~ 14.0 MHz). Coupling gel with a gel pad was placed on the skin for stand-off scanning. CPS imaging mode was used for evaluation of tumor perfusion with mechanical index of 0.25, frame rate of 5Hz, dynamic range of 78 dB, and imaging depth of 3 cm. These settings were adjusted at the beginning and maintained constant during all of the experiments.

For the ultrasound imaging studies, each mouse was anesthetized by intraperitoneal injection of pentobarbital sodium (75 mg/kg, Sigma, St. Louis, MO). Two of the 70 mice (one from group A on day 3 and one from group B from day 7) died during the anesthesia and were excluded from analysis. A heating pad was used to avoid reductions in body temperature which may affect mice blood circulation.

Initial US imaging was performed with a hand-held 7- to 14-MHz probe to find a transverse image plane containing the tumor at its maximum cross section and a large portion of the right lobe of the liver parenchyma. Before contrast agent injection, the greatest longitudinal, transverse, and anteroposterior dimensions of tumors were measured in fundamental grayscale imaging. Tumor volume was calculated using the formula for a prolate ellipsoid: volume = π/6 × length × width × depth. The maximum cross-section plane of the tumor was imaged with the transducer held manually in this position throughout the examination.

Lipid-based ultrasound contrast agent SonoVue (Bracco, Milan, Italy) dissolved with physiologic saline to 5 ml was used in this study. SonoVue was administered as a bolus (0.1 ml/20 g) into the retroorbital vein using a 27-gauge needle. The bolus injection was performed by one radiologist (W.Z., with 6 years experience in small animals study) within 1 sec for all animals to minimize variations of injection technique. Imaging was recorded on cine clips starting just before the contrast agent injection and continuing for 60 sec.

### Functional study

Off-line evaluation of the perfusion curves was performed by one investigator (Y.C.) who was blind to the treatment information. The clips were downloaded in a Digital Imaging and Communications in Medicine format for offline processing with the use of SonoTumor software (Bracco Research SA, Geneva, Switzerland) using a bolus kinetic model. Initially, a region of interest (ROI) that drawn along the margin of the tumor and a ROI at matched depth in the region of the right lobe of the liver parenchyma were selected by one investigator who was blind to the treatment information. The analysis applies first linearization at the pixel level to revert the effects of “log” compression in the ultrasound system. Results obtained from the selected ROI represented an approximately linear depiction of the backscattered intensity. The average of the linearized intensities of all the pixels in the ROI was calculated to produce a time-signal intensity curve, where the signal intensity is theoretically linked to the concentration of the microbubbles in the blood circulation [16,17], and a mathematic equation model was used to fit the contrast uptake time–intensity curve.

Four tumor perfusion parameters, including peak enhancement (PE), area under the curve of wash-in (WiAUC), wash-in rate (WiR) and wash-in perfusion index (WiPI) were calculated and normalized to the depth-matched liver parenchyma (Perfusion parameters _tumor_/Perfusion parameters _liver_). The results were noted as nPE, nWiAUC, nWiR and nWiPI (n = normalized). Quality of fit (QOF) was used to test the fit between the raw data and the fitted mathematic model. The wash-in phase of contrast was defined between the time of onset of contrast inflow and the time of peak enhancement.

### Histology

Tumors were removed and fixed in 10% buffered formalin before paraffin processing. The tumor specimens were sectioned at the largest cross sections in four-μm-thick and stained with hemotoxylin and eosin stain (H&E) to assess the cell morphology changes. Endothelial cell (CD34) density (microvascular density, MVD) was assessed by immunohistochemical method Antigen-retrieval procedure using citrate acid (pH of 6.0) was performed. Primary antibody incubation was performed using a rat antimouse CD34 antibody (clone MEC14.7, Abcam, UK) at 1:100 dilution overnight at 4°C. After rinsing with phosphate buffered saline (PBS), a secondary rabbit antirat antibody (Zhongshan Goldenbridge Biology, Beijing, China) was added and diaminobenzidine (DAB) for color development.

Regions with the highest tumor cell density in H&E stained sections were located by scanning the tissue sections under × 40-power microscope and ten different fields within the regions of highest tumor cell density were randomly chosen at × 400. The histology images of each 400× field were saved in the computer for the measurement of tumor cell density. Image pro plus software (image pro-plus 6.0; Media Cybernetics, Sliver Spring, MD, USA) was used to calculate the number of nuclei of each histology image. Data were averaged over ten fields for statistical analysis.

The measurements of MVD by counting the CD34-stained vessels under light microscopy were performed independently by two experienced observers, who were blinded to the tumor treatment and ultrasound findings according to a well established method by Weidner et al. [18]. After the “hot spots” were identified under × 40-power microscope, three fields were randomly chosen and the numbers of individual brown-stained cells were counted at × 400 powers for MVD measurements. The average of the two observers’ results was used for statistical analysis.

### Statistical analysis

All analyses were performed using SPSS version 16.0 (SPSS, Inc, Chicago, IL). The Kolmogorov-Smirnov test was applied to evaluate normal distribution. The Levene test was applied to evaluate the homogeneity of variance. One-way analysis of variance (ANOVA) tests were used to determine the significant differences of tumor volume, perfusion parameters, tumor cell density and MVD among the three groups. Confirming that there were significant differences among the three groups, the post hoc Bonferroni corrected *t* test was performed for multiple comparisons to determine difference between individual groups. The Pearson correlation test was used to determine the relationship between perfusion parameters and histopathological changes. A *P* value of < 0.05 or less was considered statistically significant.

## Results

### Effects of cisplatin treatment on tumor growth

**Figure 1:**
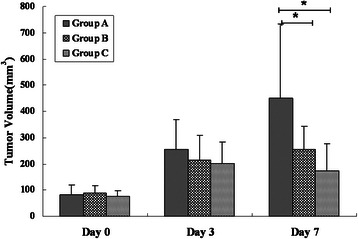
**Changes of tumor volume after treatment with different dose cisplatin.** Tumor volume of group B and C was significantly lower than that of group A on day 7 (* = *P* < 0.05) while there were no significant differences in tumor volume among the three groups on days 0, and 3 (* = *P* > 0.05). Group A, control tumors; Group B, tumors treated with 1 mg/kg cisplatin; Group C, tumors treated with 3 mg/kg cisplatin.

All mice could tolerate 7 days treatment and no obviously adverse effect was observed in the mice treated with low-dose cisplatin, however, treatment with high-dose cisplatin caused obviously adverse effects on the mice at the end of treatment, including lethargy and decrease of activity. There was no significant difference in tumor volume among the three groups on days 0 and 3 (*P* was 0.404 and 0.258, respectively). On day 7, one-way analyses showed significant difference in tumor volume among the three groups (*P* = 0.001). Tumor volume of group B and C was significantly lower than that of group A (*P* was 0.029 and 0.001, respectively), however, there was no significant difference in tumor volume between the two treated groups (*P* = 0.691) (Figure 1).

### Different dose of cisplatin treatment on tumor perfusion parameters

The raw data of bolus kinetics were well fitted to the mathematical model that the software of SonoTumor used. The mean QOF of the three groups was 0.96 ± 0.02.

In the control group (group A), CEUS demonstrated that only nPE significantly increased on day 7 as compared with day 0 (*P* = 0.023), while the other three perfusion parameters did not significantly changed on day 3 and 7 as compared with day 0 (*P* > 0.05) . In the low-dose group (group B), CEUS demonstrated that all four normalized perfusion parameters significantly decreased on day 7 as compared with day 0 (*P* < 0.05), while there were no significant changes in the perfusion parameters on day 3 as compared with day 0 (*P* > 0.05). In the high-dose group (group C), CEUS demonstrated that all four normalized perfusion parameters significantly decreased as early as 3 days after cisplatin therapy and remained low throughout the entire observation period as compared with day 0 (*P <* 0.05).

**Figure 2:**
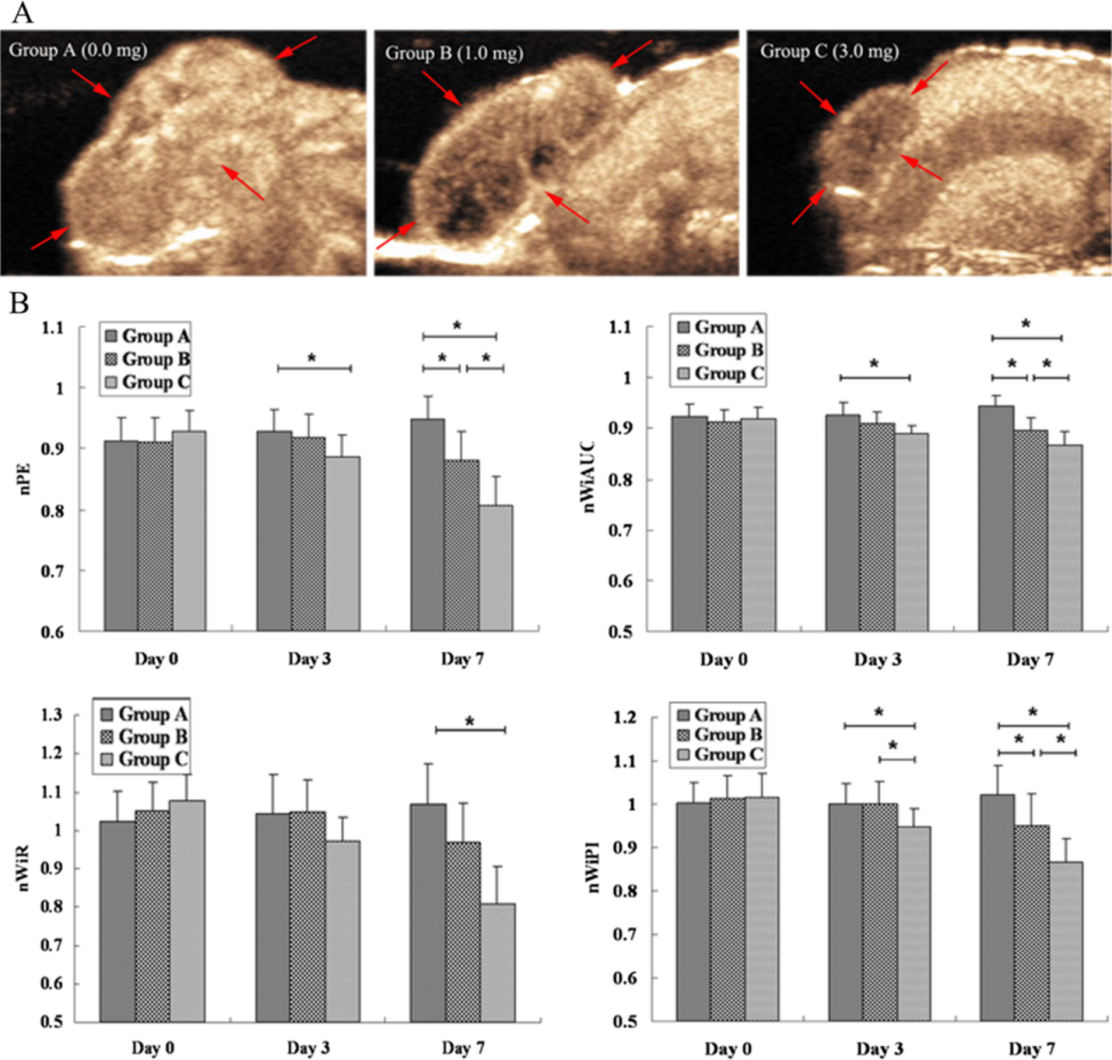
**Changes of tumor perfusion after treatment with different dose cisplatin. (A)** Representative contrast-enhanced ultrasound images of tumors (arrows) from Group A, Group B and Group C on day 7. **(B)** Changes of tumor perfusion parameters at the different time points studied. On days 3, tumor perfusion significantly decreased in group C when compared with group A (* = *P* < 0.05). On day 7, tumor perfusion significantly decreased in group B and group C when compared with group A (* = *P* < 0.05), and further decreased in group C when compared with group B (* = *P* < 0.05). Group A, control tumors; Group B, tumors treated with 1 mg/kg cisplatin; Group C, tumors treated with 3 mg/kg cisplatin.

There were no significant differences in 4 normalized perfusion parameters (i.e., nPE, nWiAUC, nWiR and nWiPI) among the three groups before treatment (*P* > 0.05). On day 3, nPE, nWiAUC and nWiPI significantly decreased in group C as compared with group A (*P <* 0.05) and only nWiPI significantly decreased in group C as compared with group B (*P* = 0.025), while there was no significant difference in perfusion parameters between group B and group A (*P* > 0.05). On day 7, nPE, nWiAUC, nWiR and nWiPI significantly decreased in group C as compared with group A (*P <* 0.05), nPE, nWiAUC and nWiPI significantly decreased in group C as compared with group B (*P* < 0.05), and nPE, nWiAUC and nWiPI significantly decreased in group B as compared with group A (*P* < 0.05) (Figure 2).

### Histopathological finding in tumors

**Figure 3:**
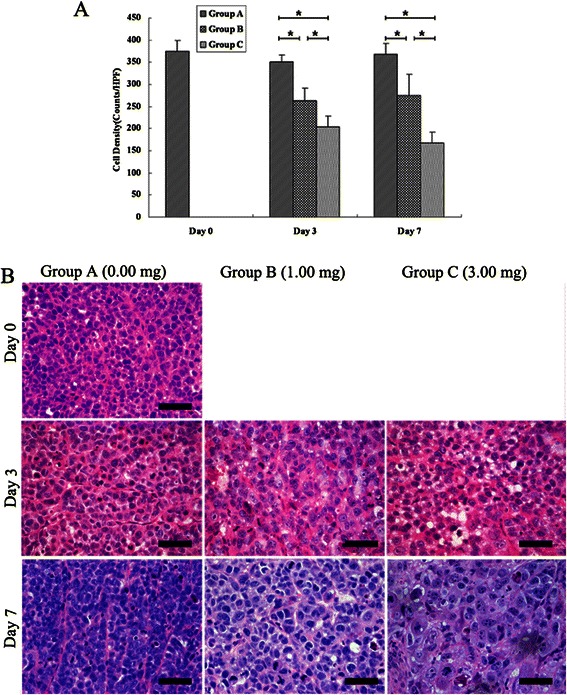
**Histopathologic analysis of tumor cell density changes after treatment with different dose cisplatin. (A)** Tumor cell density significantly decreased in groups B and C when compared with group A (* = *P* < 0.05), and further decreased in group C when compared with group B (* = *P* < 0.05). **(B)** Photomicrographs of HE stained sections of the three groups on days 0, 3, and 7. Scale bars: 50 μm.

Typical control (group A), low-dose (group B) and high-dose (group C) cisplatin-treated MCF-7 tumor sections stained by H&E are shown in Figure 3.

In the group A, tumor cell density remained stable on day 3 and 7 as compared with day 0 (*P* > 0.05). In the group B, tumor cell density significantly decreased on day 3 and 7 as compared with day 0 (*P* < 0 .001), while there was no significant difference between day 3 and 7 (*P =* 1.0). In the group C, tumor cell density significantly decreased on day 3 and 7 as compared with day 0 (*P* < 0 .001), and further decreased on day 7 as compared with day 3 (*P* = 0 .008).

On day 3 and 7, tumor cell density significantly decreased in treated tumors (groups B and C) with respect to the control tumors (group A, *P* < 0 .001) and cell density significantly decreased in group C as compared with group B (*P* < 0 .001).

In the group A, MVD remained stable on day 3 and 7 as compared with day 0 (*P* > 0.05). In the group B, MVD significantly decreased on day 7 as compared with day 0 and 3(*P* < 0 .001), while there was no significant difference between day 0 and 3(*P =* 0.437). In the group C, MVD significantly decreased on day 3 and 7 as compared with day 0 (*P* < 0 .001), while there was no significant difference between day 3 and 7 (*P =* 0.125).

**Figure 4:**
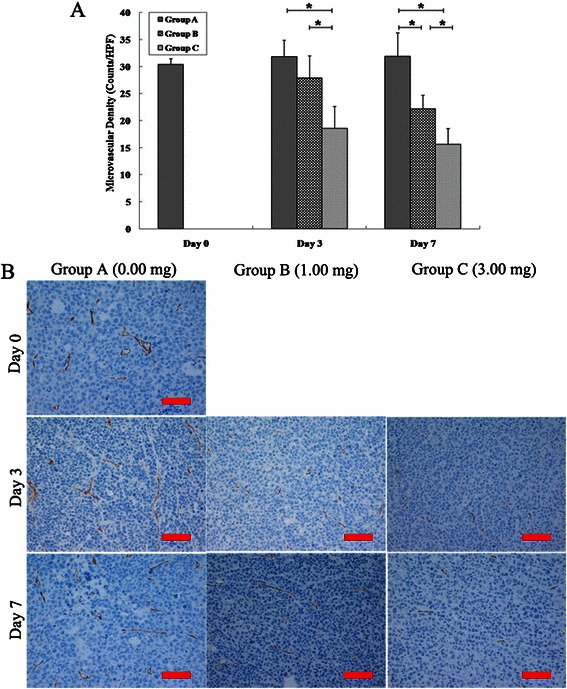
**MVD changes after treatment with different dose cisplatin. (A)** On days 3 and 7, MVD significantly decreased in group C when compared with group A and B, and further decreased in group B as compared with group A on day 7 (* = *P* < 0.05). **(B)** Photomicrographs of CD34 stained sections of the three groups on days 0, 3, and 7. Scale bars: 100 μm

On day 3, MVD significantly decreased in group C as compared with group A and B (*P* < 0 .001), while there was no significant difference in MVD between group A and B (*P* = 0.112). On day 7, MVD significantly decreased in group C as compared with group A and B (*P* < 0 .001), and MVD significantly decreased in group B as compared with group A (*P* < 0 .001) (Figure 4).

### Correlation of perfusion parameters and histopathological finding

There were positive correlations between tumor cell density and nPE (r = 0.686, *P* <0.001), nWiAUC (r = 0.681, *P* <0.001), nWiR(r = 0.606, *P* < 0.001), and nWiPI (r = 0.542, *P* <0.001). Tumor MVD stained with CD34 positively correlated with nPE (r = 0.637, *P* <0.001), nWiAUC (r = 0.604, *P* <0.001), nWiR(r = 0.633, *P* < 0.001), and nWiPI (r = 0.602, *P* <0.001).

## Discussion

Multiple strategies for improving the efficacy of chemotherapy have been explored by applying novel anti-tumor agents, different schedules, and new combination regimens. Given the theory that cells can be killed by higher doses of chemotherapy drugs but can be resistant to lower doses of the same drug, high-dose chemotherapy with some promising results became a popular yet controversial treatment [19]. It has made progress in treating certain types of tumor, however, which given serious toxic side effects. Therefore, it is important to precisely assess the response to different dose of cytotoxic therapy, so as to reduce side effects and unnecessary costs of ineffective therapy.

In this study, we evaluated the potential utility of dynamic contrast-enhanced ultrasound to provide an early index to the dosage of the chemotherapy. For this purpose, we employed MCF-7 tumor-bearing mice treated with two different dose of cisplatin (1 mg/kg.d^−1^, 3 mg/kg^−1^, i.p.) as a model system. The activity of cisplatin is thought to be a result of inter- and intrastrand DNA cross-links [20]. Therefore, cytostatic concentrations of cisplatin result in cell arrest in the G1-S or G2-M phase in vitro, and then cell death [21]. At the cisplatin doses used in this protocol (1 mg/kg and 3 mg/kg), the tumor growth was inhibited but was not completely stopped. Tumor volume of the treated and control tumors were increased on days 3 and 7 as compared with day 0 and there were no significant differences among the three groups until 7 days post treatment, tumor volumes of group B and C were significantly smaller than that of group A. In contrast, the therapeutic effectiveness of different doses cisplatin could be assessed by quantifying the tumor perfusion changes with contrast-enhanced ultrasound: treatment with higher dose cisplatin resulted in much earlier and more significant reduction in tumor blood perfusion as compared treatment with lower dose cisplatin. Moreover, the decrease in blood perfusion of treated tumors on contrast-enhanced ultrasound was associated with reduction in tumor cell density and MVD as shown by both histopathologic and immunochemistry examinations: treatment with higher dose cisplatin resulted in much earlier and more significant reduction in tumor cell density and MVD as compared treatment with lower dose cisplatin.

Due to the great need to assess tumor response to different dose of cytotoxic therapy, other imaging strategies including positron emission tomography (PET) [22], contrast-enhanced MRI [23] and contrast-enhanced CT [24], are under evaluation to determine their ability to classify tumor response to different doses of conventional chemotherapeutic drugs and antiangiogenic agents. Higher dose of chemotherapy had been associated with greater decrease in tumor glucose metabolism [25] or tumor blood perfusion [26]. However, although dynamic contrast-enhanced MRI offers relatively good sensitivity and spatial resolution in soft tissue imaging, the possibility to perform whole organ scans, as well as functional imaging [27], much of the spatial resolution must be sacrificed to get a sufficient frame rate for bolus tracking in DCE-MRI. Perfusion CT imaging is suitable for accurate assessment of tissue perfusion because change in CT image intensity is directly proportional to concentration of contrast agents [27]. However, the use of CT for repeated scanning was limited by the high concentrations of CT contrast agent and the relatively high doses of radiation. PET is highly sensitive to very low concentrations of contrast agents and is well suited to molecular imaging, however, it suffers from poor spatial resolution [27]. Ultrasound is an easy available technique for monitoring therapeutic effects and has been frequently used in clinical practice. Contrast-enhanced ultrasound imaging offers the major advantages of evaluating tumor perfusion in real time and minimal invasiveness when compared to other imaging modalities used to assess tumor blood perfusion. In this study, promising results were found: the vascular response to different dose of cytotoxic chemotherapy could be determined by using dynamic contrast-enhanced ultrasound in a mouse breast cancer model.

Conventional chemotherapeutics do not specifically target tumor cells, but rather interfere with cell division, such as by inhibiting enzymes involved in DNA replication or metabolism (for example, topoisomerases and thymidylate synthase), or microtubules. Antineoplastic drugs killing tumor cells follows first-order kinetics: specific dose kills a specific fraction of tumor cells regardless of population. In this study we found that high dose cisplatin resulted in more significant decrease in tumor cell density as compared with low dose cisplatin. Different fraction of cancer cells’ death could cause different degree decrease in tumor metabolic activity, which will correspondingly lead to different degree in the reduction of tumor blood perfusion. On the other hand, investigators have demonstrated that most conventional cytotoxic drugs can exert antiangiogenic effects [28]. Chemotherapeutics do not specifically target tumor cells, but rather interfere with cell division, including endothelial cell division takes place during new blood vessel formation [29]. So they could cause apoptosis of endothelial cells in the newly formed tumor microvessels. In this study, treated with higher dose cisplatin resulted in more significant reduction in MVD as compared treated with lower dose cisplatin and different degree of vascular disrupture could lead to different degree in the reduction of tumor perfusion.

There are some limitations in this study. First, a potential limitation of this study was that subcutaneous tumor xenografts may behave differently compared with orthotopic or transgenic mouse models, which better reflect the microenvironment of human cancers. Second, it would be of great value if quantification of tumor perfusion with contrast-enhanced ultrasound could determine the highest achievable treatment dose at which a further increasing the does of chemotherapy does not produce a further beneficial effect. However, it will be almost impossible to determine the highest achievable treatment dose, especially in an animal study, because given dose of an antineoplastic compound would destroy a certain fraction of tumor cells, and the efficiency of tumor chemotherapy, to a great extent, is dependent on the dosage of drugs and higher doses are supposed to be more effective. In this study, treatment with 3 mg/kg cisplatin resulted in much more significant decreases in tumor cell density and MVD than 1 mg/kg on the breast cancer bearing mice, however, the side effect of 3 mg/kg was much more serious than 1 mg/kg. If treatment with much higher dose, it would be questionable whether the mice could tolerate the treatment to the end of the study. Moreover, the highest achievable treatment dose should not be determined by the vascular reponse, but the relationship between tumor growth reduction and how well the patients tolerate a given dose. Further studies are needed to determine the relationships between tumor vascular response and the longitudinal treatment outcome. Third, although there were positive correlations between US perfusion parameters and the histopathological findings (cell density and MVD), the changes in cell density and MVD were more profound than those in US perfusion parameters, which might due to that cell density and MVD were measured from the “hot spots” approach, and US perfusion data was measured from the tumor of maximum cross section. A complete quantification of tumor perfusion and histopathological changes throughout the whole tumor volume could improve the relevance of the US perfusion parameters with cell density and MVD.

## Conclusions

In conclusion, our study suggests that contrast-enhanced US by quantifying intratumor blood flow can evaluate tumor vascular response to different dose of chemotherapy, which may help to guide drug dosage during tumor chemotherapy.
